# Polymer Coated CaAl-Layered Double Hydroxide Nanomaterials for Potential Calcium Supplement

**DOI:** 10.3390/ijms151222563

**Published:** 2014-12-05

**Authors:** Tae-Hyun Kim, Jeong-A Lee, Soo-Jin Choi, Jae-Min Oh

**Affiliations:** 1 Department of Chemistry and Medical Chemistry, College of Science and Technology, Yonsei University, Wonju, Gangwondo 220710, Korea; E-Mail: th.kim@yonsei.ac.kr; 2 Department of Food Science and Technology, Seoul Women’s University, Seoul 139774, Korea; E-Mail: junga0462@hanmail.net

**Keywords:** CaAl-LDH, polymer coating, pharmacokinetics, toxicity

## Abstract

We have successfully prepared layered double hydroxide (LDH) nanomaterials containing calcium and aluminum ions in the framework (CaAl-LDH). The surface of CaAl-LDH was coated with enteric polymer, Eudragit^®^L 100 in order to protect nanomaterials from fast dissolution under gastric condition of pH 1.2. The X-ray diffraction patterns, Fourier transform infrared spectroscopy, scanning electron and transmission electron microscopy revealed that the pristine LDH was well prepared having hydrocalumite structure, and that the polymer effectively coated the surface of LDH without disturbing structure. From thermal analysis, it was determined that only a small amount (less than 1%) of polymer was coated on the LDH surface. Metal dissolution from LDH nanomaterials was significantly reduced upon Eudragit^®^L 100 coating at pH 1.2, 6.8 and 7.4, which simulates gastric, enteric and plasma conditions, respectively, and the dissolution effect was the most suppressed at pH 1.2. The LDH nanomaterials did not exhibit any significant cytotoxicity up to 1000 μg/mL and intracellular calcium concentration significantly increased in LDH-treated human intestinal cells. Pharmacokinetic study demonstrated absorption efficiency of Eudragit^®^L 100 coated LDH following oral administration to rats. Moreover, the LDH nanomaterials did not cause acute toxic effect *in vivo*. All the results suggest the great potential of CaAl-LDH nanomaterials as a calcium supplement.

## 1. Introduction

Along with the rapid development of nanotechnology in diverse industrial fields, nanomaterials have been also widely applied to bio-related products, such as medicine, medical devices, pharmaceutics, personal care products, food *etc.* to endow a novel physicochemical property, to enhance functionality, bioactivity and bioavailability, and to effectively deliver bioactive molecules to target specific organs [1,2,3,4]. Application of nanomaterials in human-related biological fields can be generally categorized into several sectors: (1) nanomedicines including drug delivery systems or diagnostics; (2) consumer products like UV-screening agents; (3) food additives such as anticaking and whitening agents or nutritional supplements to fortify absorption of nutrients [5,6,7]. Currently, most studies on nano-bio materials have been done in order to enhance stability and absorption of bioactive substances by encapsulating them in nanomaterials [8,9,10,11,12]. However, attempts on the development of functional nanomaterial itself as a nutritional supplement have not been extensively carried out.

Calcium is one of the essential inorganic elements in biological systems not only as a major constituent of skeletal systems, such as bone and teeth, but also as a mediator in various physiological functions [13,14,15]. It has been reported that the calcium deficiency results in serious diseases, such as osteoporosis and hypocalcaemia. Hence, various calcium supplements including calcium carbonate, calcium citrate, calcium phosphate and calcium gluconate have been developed [16]. Among them, calcium carbonate obtained from oyster shell is widely distributed on the market, because it is relatively inexpensive and abundant in nature [17]. However, it is generally known that oral bioavailability of calcium from calcium carbonate supplement is extremely low, approximately 4% [18].

As a potential candidate for calcium supplement nanomaterials, we developed layered double hydroxide (LDH) nanoparticle, a kind of layered mineral. LDHs are composed of positively charged mixed metal hydroxide layers and exchangeable interlayer anions. The general formula of LDHs is represented as follow: [M(II)_1-x_M(III)_x_(OH)_2_]^x+^(A^n−^)_x/n_·mH_2_O (M: divalent or trivalent metal ions, A^n−^: anionic species). Due to the diversity of exchangeable anions, intercalation capacity and biological inertness, LDHs have been explored as delivery nanovehicles for drugs and bioactive molecules [19,20]. Furthermore, LDHs have been reported to enhance cellular uptake rate as well as chemical/biological stability of intercalated biomolecules [21,22]. Calcium cations are known to readily dissolve in acidic and neutral pH, and then form hydroxide precipitates in basic conditions. Thus, it is expected that calcium containing LDHs are expected to be dissolved into absorbable ions under gastric (pH~1.2) and small intestine (pH~6.8) conditions. Incorporation of bioactive molecules into calcium containing LDHs further enables the development of a dual functional system having both calcium supplement and delivery functions. 

In this paper, we prepared calcium containing LDH nanomaterials, CaAl-LDH, and the surface of calcium-containing LDH was then coated with enteric coating polymer, Eudragit^®^L 100 [23], aiming to facilitate efficient calcium dissolution and absorption in the small intestine [24]. The Eudragit^®^L 100 polymer consisting of methacryalate and metharcylic acid is known to flocculate at acidic pH and be solubilized at pH above ~6.0, an intestinal pH condition [25]. Hence, the Eudragit^®^L 100 coating can protect LDH from acidic gastric conditions, and also help to dissolve LDH in small intestine. Physicochemical properties and pH-dependent solubility of Eudragit^®^L 100-coated CaAl-LDH (E-coated CaAl-LDH) were characterized. Furthermore, its cellular calcium uptake, pharmacokinetics and *in vitro* and *in vivo* toxicity were evaluated.

## 2. Results and Discussion

**Figure 1:**
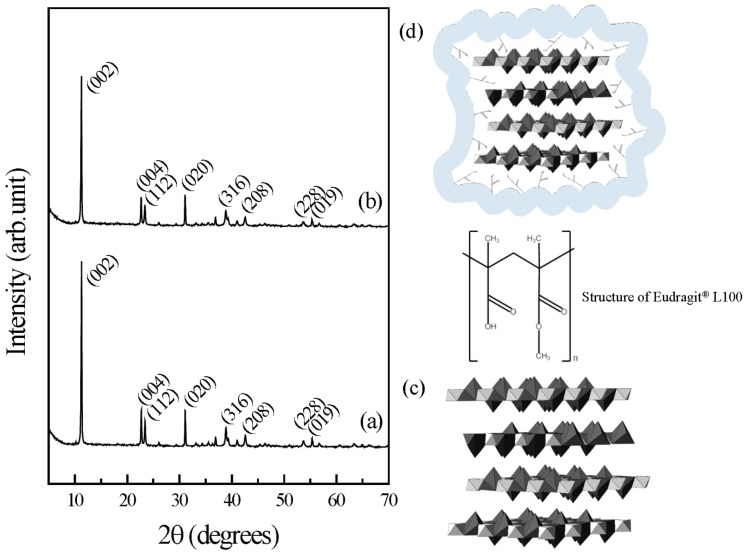
Powder XRD patterns for (**a**) pristine CaAl-LDH and (**b**) E-coated CaAl-LDH; (**c**) Crystal structure of CaAl-LDH (hydrocalumite, JCPDS No. 31-0245) and (**d**) schematic illustration of E-coated CaAl-LDH.

The crystal structure of pristine CaAl-LDH was confirmed with powder X-ray diffraction (XRD) patterns (Figure 1). As the LDH consisted of the stacking of 2-dimensional metal hydroxide nanosheets along the *z*-axis, we observed intense (002) and (004) peaks in the diffraction patterns. We could assign the characteristic diffraction peaks of Ca-containing LDH to (112), (020) and (316) at 2-theta values of 23.4°, 31.1° and 38.9° of hydrocalumite (Ca_4_Al_2_O_6_Cl_2_·12H_2_O, JCPDS No 31-0245), respectively. The calculated lattice parameters, a_0_ and c_0_ of 11.51 and 17.10 Å, respectively, also reflected that the obtained pristine LDH has the expected structure as previously reported [26]. 

As the peak positions and patterns of E-coated CaAl-LDH were almost same to those of pristine LDH, we could confirm that the coating process was successfully carried out without disturbing the overall crystal structure of LDH. In order to investigate the crystallinity change of CaAl-LDH upon Eudragit^®^L 100 coating, we calculated the crystallite sizes of samples based on Scherrer’s equation (Equation (1)) [27] as to two major peaks, (002) and (020). Crystallite sizes of CaAl-LDH calculated from (002) and (020) peaks were 48.4 and 52.6 nm, respectively, while those values of E-coated CaAl-LDH were 45.8 and 48.3 nm, respectively. Accordingly, crystallite size was reduced by ~5% and ~8% in (002) and (020) direction during coating process. This decrement might be attributed to slight dissolution of pristine LDH during coating process. However, the degree of dissolution seems to be low to not cause remarkable loss of pristine LDH during the coating process.

(1) $$t=\frac{0.9\text{λ}}{\beta \text{cos}\theta }$$

*t*: crystallite size; λ: X-ray wavelength of 1.5405 Å; β: Full width at half maximum of peak; θ: Bragg angle.

Surface coating of LDHs with Eudragit^®^L 100 was performed to protect them from acidic condition, not to modify their chemical structure. It is worth to note that we used ethanol rather than alkaline solution to solubilize Eudragit^®^L 100 for the coating process. The alkaline solution can solubilize Eudragit^®^L 100 through detaching protons from methacrylic acid residue, thereby inducing a negative charge at the polymer side chain. It was reported that the polymer could be intercalated into the interlayer space of layered materials when they possessed strong and periodic negative charge on the backbone, resulting in the formation of polymer-inorganic layer-by-layer hybrid structure or swelling of the layered structure [28,29,30,31]. Therefore, the methacrylic acid residues could be preserved in ethanol solution and gradually interact with the basic surface of LDHs. In this way, Eudragit^®^L 100 could coat homogeneously the LDH surface without intercalation. 

**Figure 2:**
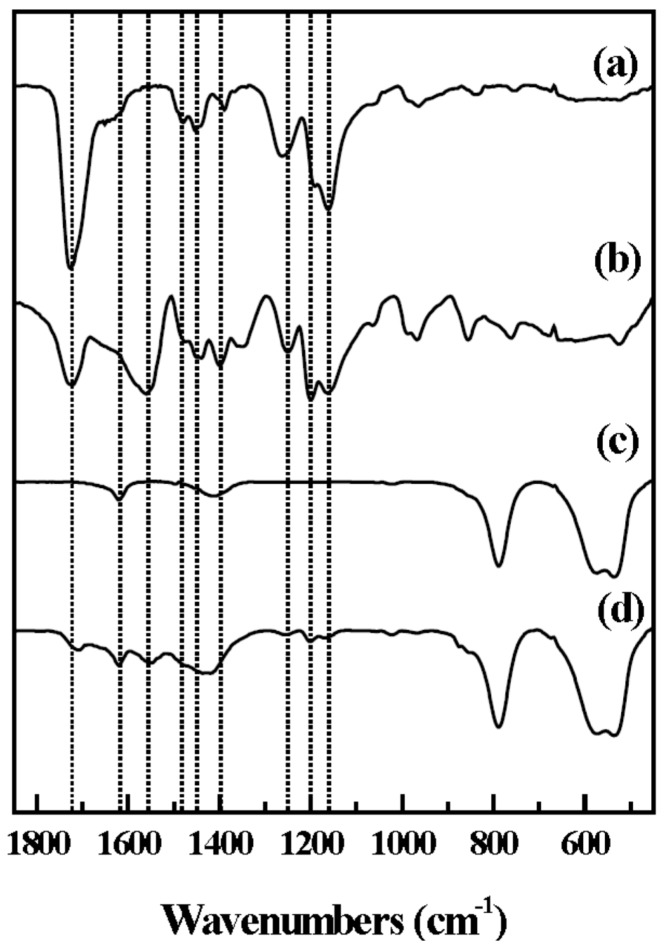
FT-IR spectra of (a) Eudragit^®^L 100 (b) Na^+^-Eudragit^®^L 100 salt (c) pristine CaAl-LDH and (d) E-coated CaAl-LDH. The dotted lines are positioned and 1727, 1625, 1556, 1482, 1448, 1392, 1252, 1195, and 1166 cm^−1^, respectively.

The surface coating of LDH with Eudragit^®^L 100 was also verified by Fourier transform infrared (FT-IR) spectra (Figure 2). We observed characteristic stretching vibration of Eudragit^®^L 100 in the spectrum of coated LDHs; peaks at 1482 and 1445 cm^−1^ were attributed to CH_x_ stretching and the peaks at 1252, 1195 and 1166 cm^−1^ are attributed to the ester bond in methacrylate residue. It should be noted that the COO^−^ asymmetric and symmetric stretching vibrations at 1556 and 1392 cm^−1^ were observed in E-coated CaAl-LDH (Figure 2d), which were not found in the spectrum of Eudragit^®^L 100 only (Figure 2a). It is suggested that during the coating process, the polymer interacted with the basic surface of LDH, resulting in partial deprotonation, and then the carboxylate residues attached on the positive LDH surface through electrostatic interaction. The percentage of ionic bonding (PIB) was calculated by comparing the vibration energy of ν_(COOH)_, ν_(COOM)_, and ν_(COONa)_ (M stands for a counter ion and corresponds to the CaAl-LDH in this study, Equation (2)) [32].

PIB = [ν_(COOH)_ − ν_(COOM)_]/[ν_(COOH)_ − ν_(COONa)_] (2)

The PIB value for E-coated CaAl-LDH was determined to be 1.06, which means that the bond between Eudragit^®^L 100 and LDH was strong enough compared with the ionic one. This result suggests that the polymer well covered the surface of LDH nanomaterials through strong electrostatic interaction.

**Figure 3:**
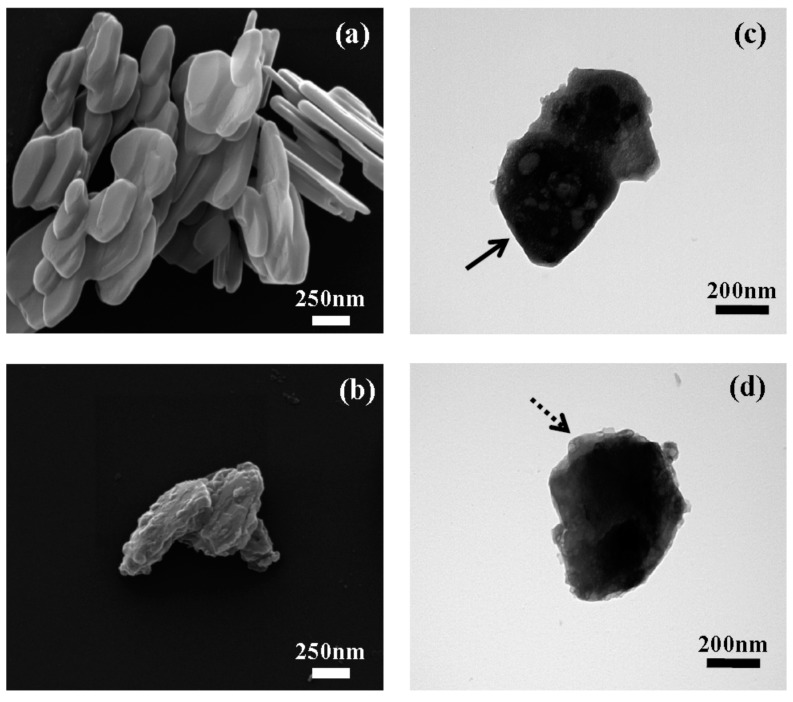
SEM images of (**a**) pristine CaAl-LDH and (**b**) E-coated CaAl-LDH. TEM images of (**c**) pristine CaAl-LDH and (**d**) E-coated CaAl-LDH. The arrow in (**c**) and the dashed arrow in (**d**) stand for the sharp edge of pristine LDH and blunt part of E-coated CaAl-LDH, respectively.

The particle size and morphology of both pristine CaAl-LDH and E-coated CaAl-LDH were examined with scanning electron microscopy (SEM) and transmission electron microscopy (TEM) (Figure 3). The SEM images showed that both CaAl-LDH (before and after coating) had a typical plate-like morphology with particle size distribution ranging from 200 to 500 nm, indicating the average size being preserved throughout the coating process. However, we found a slight morphological change between pristine and coated LDH nanomaterials; the pristine LDH exhibits smooth and flat surface, while the surface of polymer coated LDH became bumpy. The random assembly of polymer chains on the LDH surface was thought to induce a rough surface and blunt edges. According to the TEM analysis, we obtained similar results. TEM images of pristine CaAl-LDH showed primary particle size of approximately 300 nm with sharp edges (arrow in Figure 3c), whereas, the image for E-coated CaAl-LDH showed dark contrast at center with gray contrast at the edge (dashed arrow in Figure 3d). The blunt morphology with gray contrast in E-coated CaAl-LDH was considered as thin layers of polymer coated on inorganic surface.

**Figure 4:**
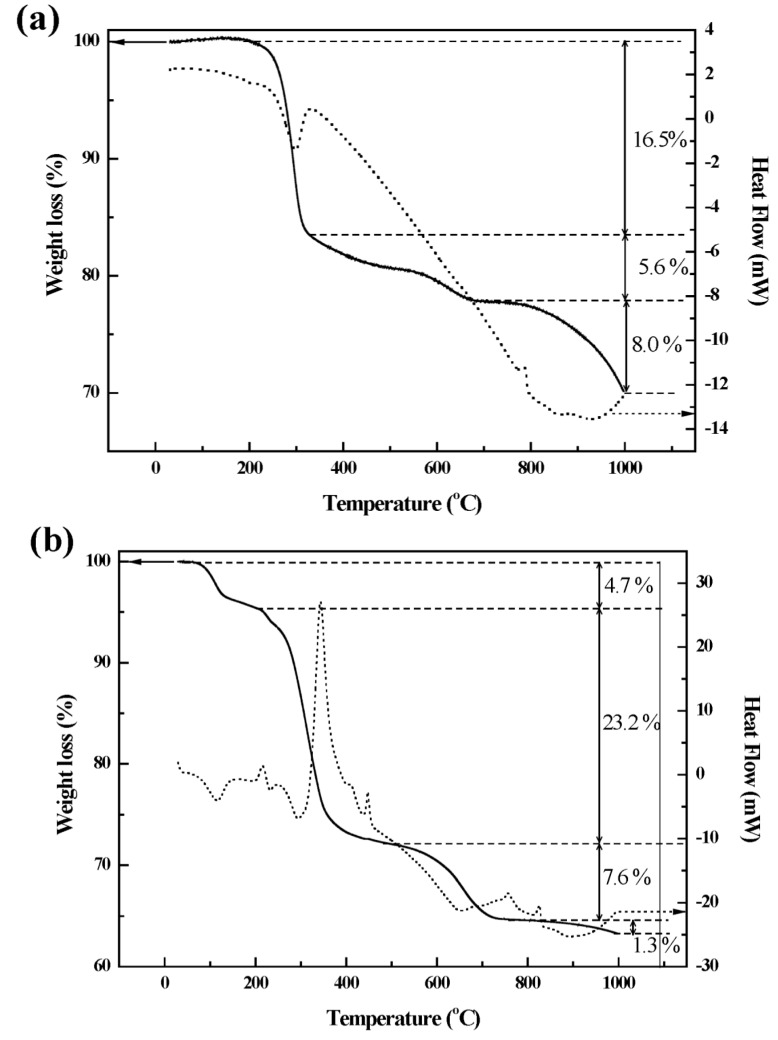
TG and DSC curves for (**a**) pristine CaAl-LDH and (**b**) E-coated CaAl-LDH. Solid line: TG; dotted line: DSC.

We further evaluated the chemical formula of the prepared samples with inductively coupled plasma-atomic emission spectroscopy (ICP-AES) and thermal analyses. From ICP-AES analysis, the metal ratios of Ca:Al for the two samples were determined to be 2.05:1 and 2.07:1, respectively, revealing that there was almost no dissolution of LDH nanomaterials during the polymer coating process. For thermogravimetric (TG) and differential scanning calorimetry (DSC) analysis, the two samples were pre-heated at 100 °C to remove surface adsorbed water. In the TG curve for pristine LDH (Figure 4a), we observed three steps of weight loss. The first weight loss of ~16.5% with an endothermic peak at around 300 °C was attributed to partial dehydroxylation of the LDH layers [33,34]. The second and third weight loss ranging from 490 to 1000 °C were attributed to be the decomposition of hydroxides and the elimination of interlayer anion. The E-coated CaAl-LDH exhibited similar thermal behavior. The second step ranging from 200 to 485 °C was due to the partial dehydroxylation of the LDH layer combined with thermal decomposition of coated Eudragit^®^ L-100. This step showed increased weight loss (23.2%) compared with the pristine (18.6%) and strong exothermic peak at 320 °C, which confirmed that coated polymer combusted during this step. The third and fourth weight losses in the region from 485 to 1000 °C were attributed to the full dehydroxylation and elimination of interlayer anions. Based on the TG result, chemical composition of E-coated CaAl-LDH was determined to be [Ca_2.07_Al(OH)_6.14_Cl][Eudragit^®^ L-100]_0.013_, which represents the polymer contents of 0.85% (*w*/*w*) in the coated sample.

Ca^2+^ dissolution from LDH lattice with respect to time and pH conditions was also evaluated. We prepared three different aqueous solutions with pH values of 1.2, 6.8 and 7.4 in order to simulate gastric, enteric and plasma pH conditions, respectively. It is generally known that calcium makes hydroxide precipitates at highly basic condition (pH > 11) and their solubility is accelerated with decreasing pH. Thus, it was expected that CaAl-LDH is dissolved into ionic species at all the simulated pH conditions, with maximum dissolution at pH 1.2 (gastric condition). We measured metal ion concentrations in the supernatant of each suspension and calculated the dissolved weight percentage of metal ions from the LDH lattice. As expected, about 80% of pristine LDH was dissolved into ionic species at pH 1.2 (Table 1), and less dissolution was found at higher pH of 6.8 (Table 2) and 7.4 (Table 3), showing approximate dissolution of 50%. The pH dependent dissolution pattern of E-coated CaAl-LDH showed significant difference compared with the pristine LDH. The overall dissolution of polymer coated LDH was suppressed, showing dissolution ratio of ~60%, ~40% and ~38% at pH 1.2, 6.8 and 7.4, respectively. The dissolution of metal ions from E-coated CaAl-LDH was highly suppressed in acidic pH, indicating that the polymer coating effectively protected LDH nanomaterials from acidic condition. Therefore, E-coated CaAl-LDH can be applied as a calcium supplement, targeting calcium absorption in the small intestine (pH~6.8). The solubility of commercially available food grade calcium carbonate obtained from seashell was determined to be approximately 0.7 wt % in pH 1.2 solution and less than 0.01 wt % in pH 6.8 and 7.4 solutions. In terms of Ca^2+^ dissolution under intestinal condition, E-coated CaAl-LDH is expected to have higher bioavailability than commercial calcium carbonates.

**Table 1:** Time dependent Ca^2+^ dissolution of CaAl-LDH and E-coated CaAl-LDH at pH 1.2.

pH 1.2	Concentration (ppm)				Dissolution Percentage (wt %)			
Material	Pristine LDH		Coated LDH		Pristine LDH		Coated LDH	
Metal Time	Ca^2+^	Al^3+^	Ca^2+^	Al^3+^	Ca^2+^	Al^3+^	Ca^2+^	Al^3+^
30 min	10.8	3.31	6.49	2.02	82.20	76.72	52.09	49.38
120 min	11.4	3.48	7.57	2.39	86.77	80.66	60.76	58.42
480 min	11.0	3.53	8.57	2.77	83.72	81.82	68.79	67.71

**Table 2:** Time dependent Ca^2+^ dissolution of CaAl-LDH and E-coated CaAl-LDH at pH 6.8.

pH 6.8	Concentration (ppm)				Dissolution Percentage (wt %)			
Material	Pristine LDH		Coated LDH		Pristine LDH		Coated LDH	
Metal Time	Ca^2+^	Al^3+^	Ca^2+^	Al^3+^	Ca^2+^	Al^3+^	Ca^2+^	Al^3+^
30 min	5.86	1.93	4.93	1.63	44.60	44.73	39.57	39.84
120 min	6.08	2.01	5.2	1.67	46.28	46.59	41.74	40.82
480 min	6.37	2.49	5.03	1.78	48.48	57.71	40.38	43.51

**Table 3:** Time dependent Ca^2+^ dissolution of CaAl-LDH and E-coated CaAl-LDH at pH 7.4.

pH 7.4	Concentration (ppm)				Dissolution Percentage (wt %)			
Material	Pristine LDH		Coated LDH		Pristine LDH		Coated LDH	
Metal Time	Ca^2+^	Al^3+^	Ca^2+^	Al^3+^	Ca^2+^	Al^3+^	Ca^2+^	Al^3+^
30 min	N.D	N.D	4.13	1.04	N.D	N.D	33.15	25.42
120 min	6.13	2.10	4.81	1.61	46.66	48.67	38.61	39.35
480 min	6.45	2.64	5.05	2.18	49.09	61.19	40.54	53.29

**Figure 5:**
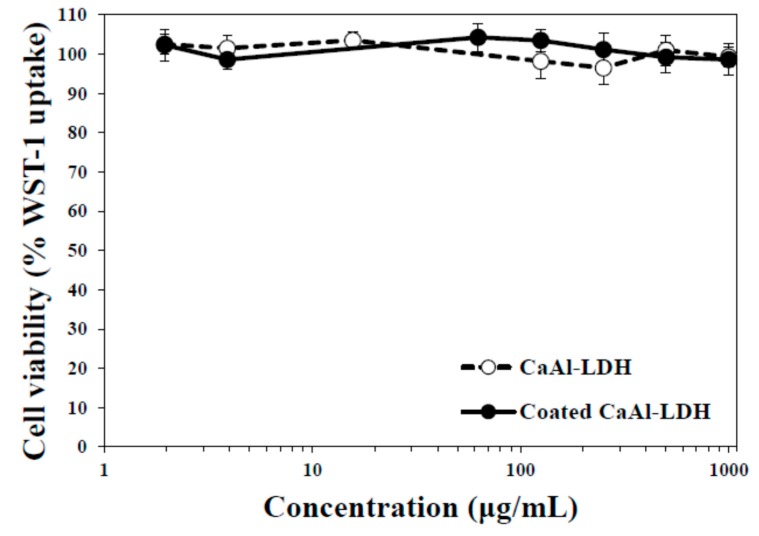
Cell viability of CaAl-LDH treated CaCo-2 cells after 72 h incubation, measured by water-soluble tetrazolium (WST)-1 assay.

To be applied as a food supplement, biocompatibility of nanomaterials is also an important factor. Hence, cytotoxicity of CaAl-LDH and E-coated CaAl-LDH was evaluated in human epithelial colorectal CaCo-2 cells. As shown in Figure 5, both nanoparticles did not exhibit cytotoxic effect up to the highest concentration tested, 1000 μg/mL, after 72 h incubation. The low cytotoxicity of CaAl-LDH is in good agreement with our previous reports [35,36], showing little effect of LDHs on cell proliferation and viability of cells. 

**Figure 6:**
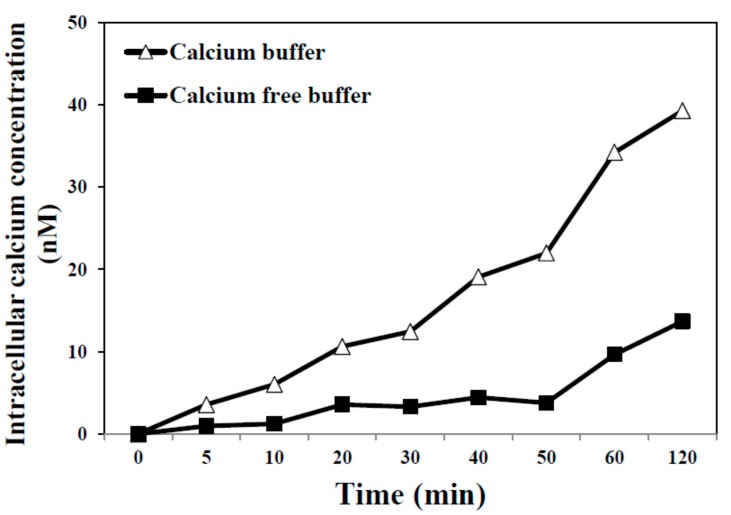
Intracellular calcium levels in CaCo-2 cells exposed to CaAl-LDH nanomaterials, measured by Calcium Green™-1.

Intracellular Ca^2+^ amount in CaAl-LDH exposed cells was traced with a cell permeant fluorescent probe, Calcium Green™-1, which increases green fluorescence upon binding Ca^2+^. Figure 6 showed that calcium concentration increased in CaAl-LDH treated cells as incubation time increased. Interestingly, intracellular calcium level dramatically increased in calcium containing buffer, as compared with that in calcium free buffer. This result implies that nanomaterials interact strongly with Ca^2+^, probably forming Ca^2+^-CaAl-LDH complex, which facilitates free Ca^2+^ uptake into cells. On the other hand, increased calcium level in calcium free buffer directly indicates intracellular uptake amount of CaAl-LDH into cells. This might be closely associated with the fact that LDH nanoparticles can be internalized into cells via the endocytosis pathway, as particulate forms [37]. Significantly increased calcium levels was not found in cells incubated with commercially available food grade calcium carbonate (data not shown), probably related to its bulk size.

**Figure 7:**
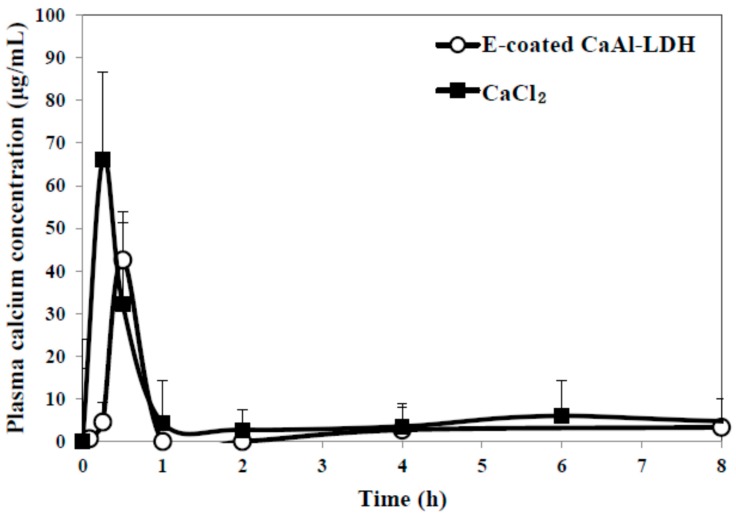
Plasma concentration-time curve of E-coated CaAl-LDH after a single dose oral administration to rats. Equivalent amount of CaCl_2_ based on calcium content was also treated as a control group.

Pharmacokinetics of nanoparticles was evaluated after a single dose oral administration to rats, in comparison with CaCl_2_ as a calcium ion reference (Figure 7). Plasma concentration-time curve of E-coated CaAl-LDH demonstrated that calcium concentration rapidly increased, showing peak concentration at 0.5 h, and decreased within 1 h. On the other hand, calcium ions were determined to be more rapidly and highly absorbed into the blood stream. When pharmacokinetic parameters between E-coated CaAl-LDH and calcium ion were compared (Table 4), high C_max_ and AUC values for calcium ions were found, indicating high absorption amount. However, when absorption efficiency (%) was considered, calculated by total plasma calcium concentration divided by administered amount, high oral absorption efficiency (about 8%) was found for E-coated CaAl-LDH. This is because calcium content in LDH nanomaterials is much lower than that in CaCl_2_, suggesting that E-coated CaAl-LDH was effective to enhance oral calcium absorption. It is worthy to note that oral absorption efficiency of commercially available food grade calcium carbonate was about 4% (data not shown), which is highly consistent with its known oral absorption [18]. 

**Table 4:** Pharmacokinetic parameters of E-coated CaAl-LDH, as compared with CaCl_2_ after a single dose oral administration to rats.

Pharmacokinetic Parameters	E-Coated CaAl-LDH	CaCl_2_
C_max_ (μg/mL)	42.55 ± 6.14 ^a^	66.16 ± 12.98 ^b^
T_max_ (h)	0.5	0.25
AUC (h × μg/mL)	31.56 ± 0.62 ^a^	39.19 ± 5.74 ^b^
T_1/2_ (h)	0.65 ± 0.05 ^a^	0.77 ± 0.04 ^b^
MRT (h)	1.24 ± 0.06 ^b^	0.97 ± 0.09 ^a^
Absorption (%) *	8.13 ± 0.16 ^b^	3.05 ± 0.45 ^a^

^a,b^ Significant difference between E-coated CaAl-LDH and CaCl_2_; * Absorption was calculated based on AUC values.

**Figure 8:**
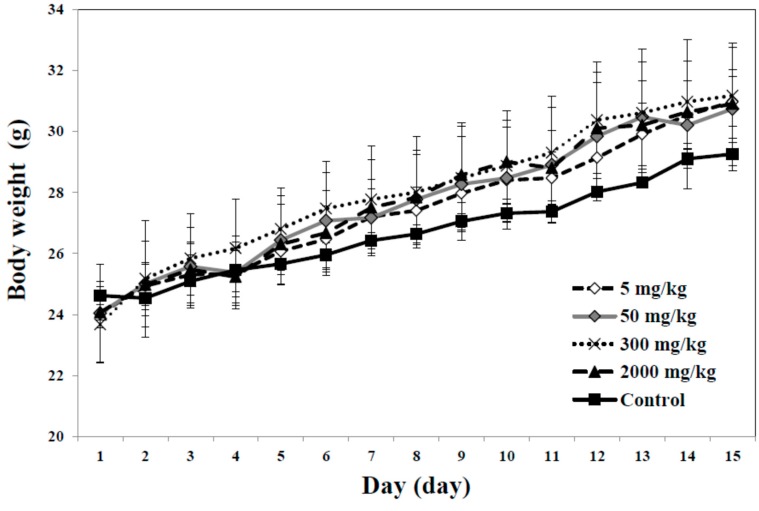
Change in body weight in mice after a single dose oral administration of E-coated CaAl-LDH.

Finally, acute toxicity of E-coated CaAl-LDH was evaluated after a single dose oral administration to mice. No mortality, significant decrease in body weight and abnormal behaviors or symptoms were observed up to the dose of 2000 mg/kg (Figure 8). This result clearly demonstrated low acute toxicity of the present nanoparticles, suggesting their promising potential as a calcium supplement. All the results suggest that E-coated CaAl-LDH can be effective to enhance oral absorption and bioavailability of calcium at the systemic level.

## 3. Experimental Section

### 3.1. Preparation of Ca-Containing LDH

CaCl_2_·2H_2_O and NaOH were obtained from Daejung Chemicals and Metals Co., Ltd. (Siheung-si, Gyonggi-do, Korea), AlCl_3_·6H_2_O was purchased from Sigma Aldrich Co., Ltd. (St. Louis, MO, USA) and Eudragit^®^L 100 was purchased from Evonik Industries (Essen, North Rhine-Westphalia, Germany). Ethyl alcohol (95%) was obtained from SK Chemicals (Seongnam-si, Gyonggi-do, Korea). All the reagents were used without further purification. Food grade calcium carbonates, produced by seashells, were purchased from Apexel. Co., Ltd. (Pohang-si, Gyeongsangbuk-do, Korea). 

LDH consisted of calcium and aluminum (CaAl-LDH) was prepared through coprecipitation method, as previously reported [38]. Typically, NaOH solution (1.26 M) was added dropwise to the mixed metal chloride solution (0.315 M and 0.1575 M for CaCl_2_·2H_2_O and AlCl_3_·6H_2_O, respectively) until the pH reached to around 11.5. The obtained white suspension was aged for 24 h at room temperature with vigorous stirring. Then, the resulting precipitates were filtered and washed thoroughly with deionized water and dried in a vacuum at 40 °C.

### 3.2. Coating of Nanoparticles with an Enteric Polymer

In order to coat the surface of LDH with Eudragit^®^ L100 (E-coated CaAl-LDH), the polymer (0.247 g) was dissolved in ethanol (250 mL). Then, 1 g of CaAl-LDH was dispersed into 250 mL ethanol and Eudragit^®^L 100 solution was added dropwise. After stirring the suspension for 6 h, the precipitate was obtained by filtration and dried in a vacuum at 40 °C.

### 3.3. Physicochemical Characterization

Powder XRD of pristine CaAl-LDH and E-coated CaAl-LDH were obtained with Bruker AXS D2 phaser with increments of degree and time step of 0.02° and 1 sec/step, respectively. FT-IR (Spectrum one B.v5.0, Perkin Elmer, Waltham, MA, USA) spectroscopy was performed with conventional KBr methods. The chemical composition and thermal behavior of all prepared samples were obtained with ICP-AES (Optima-4300 DV, Perkin Elmer, Waltham, MA, USA), TG (SDT-Q600, TA Instruments, New Castle, PA, USA) and DSC (SDT-Q600, TA Instruments, New Castle, PA, USA). The particle size and morphology of prepared samples were observed with TEM (JEM1010 at National instrumentation center for environmental management in Seoul National University, Korea) and SEM (Quanta 250 FEG at Yonsei University in Wonju, Korea).

### 3.4. Dissolution Test 

Dissolution of Ca^2+^ from either pristine CaAl-LDH or E-coated CaAl-LDH was evaluated in three different aqueous solutions with pH at 1.2, 6.8, and 7.4, simulating gastric, enteric and plasma pH conditions, respectively. To evaluate the pH dependent dissolution of nanomaterials, only pH was controlled without addition of enzyme or salt. Either CaAl-LDH or E-coated CaAl-LDH (200 mg, respectively) was dispersed in each simulated solution. At time points of 0.5, 2 and 8 h, 2 mL of solution was collected and the supernatant was massed up to 50 mL for ICP-AES analysis (Optima-4300 DV, Perkin Elmer, Waltham, MA, USA).

### 3.5. Cell Viability

Human epithelial colorectal adenocarcinoma CaCo-2 cells were purchased from the Korean Cell Line Bank and cultured in Minimum Essential Media (MEM, Welgene, Ltd., Daegu, Korea), under a humidified atmosphere (5% CO_2_ plus 95% air) at 37 °C. The medium was supplemented with 10% heat inactivated fetal bovine serum (Welgene Ltd., Daegu, Korea), 100 units/mL penicillin and 100 µg/mL streptomycin. Cells (5 × 10^3^ cells/100 µL) were seeded onto 96-well plates and incubated overnight at 37 °C in 5% CO_2_ atmosphere. The medium in the wells was then replaced with fresh medium containing nanoparticles (1–1000 µg/mL) and incubation continued for 72 h. The effect of the nanoparticles on cell proliferation was determined by WST-1 assay (Roche Ltd., Basel, Switzerland). Briefly, 10 µL of WST-1 solution (Roche) was added to each well and the plates were further incubated. After 4 h, the absorbance was measured with a plate reader at 440 nm. Cells incubated without nanomaterials were used as a control.

### 3.6. Intracellular Uptake 

Intracellular uptake of Ca^2+^ from nanomaterials was measured with Calcium Green™-1 (Molecular Probes, Eugene, OR, USA). Cells were grown at 5 × 10^4^ cells/96 well and then incubated with CaAl-LDH nanoparticles at 250 μg/mL in the presence of 2 μM Calcium Green™-1 in Krebs-Ringer-HEPES solution (KRH, American Research Products, Inc., Waltham, MA, USA) containing (mM/L) NaCl 125.0, KCl 5.0, KH_2_PO_4_ 1.2, CaCl_2_ 2.0, MgSO_4_ 1.2, glucose 6.0 and HEPES 25.0, and adjusted to pH 7.4. For experiments requiring calcium-free solution, CaCl_2_ was omitted from KRH solution and 1 mM EDTA was added to complex any trace of Ca^2+^ contaminant. Fluorescence intensity of the Ca^2+^-bound indicator was measured with a fluorescence microplate reader (Dynex Technology Inc., Chantilly, VA, USA), with excitation at 506 nm and emission at 531 nm. The amount of intracellular Ca^2+^ was calculated by performing a calibration with external standards of calcium and Green™-1, according to the manufacturer’s protocol.

### 3.7. Pharmacokinetics

Male SD rats, aged 5 weeks and weighing 180–200 g, were purchased from the G-Bio (Gyeonggi-Do, Korea). The animals were housed in plastic lab animal cages in a ventilated room. The room was maintained at 20 ± 2 °C and 60% ± 10% relative humidity with a 12 h light/dark cycle. Water and commercial laboratory complete food for mice were available *ad libitum*. They were acclimated to this environment for 7 days before treatment. All animal experiments were performed in compliance with the Animal and Ethics Review Committee of the Seoul Women’s University.

The plasma concentration of calcium was analyzed after oral administration of E-coated CaAl-LDH or calcium chloride (CaCl_2_) as a control to each group of six male rats. Four mg of calcium based on calcium content in E-coated CaAl-LDH or CaCl_2_ were administered via oral gavage. The blood samples (about 0.2 mL) were collected via tail vein at several time points (0, 15 and 30 min, 1, 2, 4, 6, and 8 h). The blood sample at 0 h before oral administration was used to determine the basal calcium level in the plasma. The blood samples were centrifuged at 3000 rpm for 15 min at 4 °C to obtain the plasma and stored at −70 °C before analysis.

The plasma was digested in 3 mL of ultrapure nitric acid overnight, heated at about 160 °C, and then, 0.5 mL of H_2_O_2_ was added. Each sample mixture was heated until completely digested. The remaining solution was then removed by heating until the solutions were colorless and clear. The solution were finally diluted to 5 mL with ultrapure water and filtered with a 0.45 μm pore filter. The total calcium content was determined using ICP-AES (Jobin Yvon Horiba, JY2000 Ultrace, Longjumeau, France). The following pharmacokinetic parameters were estimated using Kinetica version 4.4 (Thermo Electron Corporation, Waltham, MA, USA): maximum concentration (C_max_), time to maximum concentration (T_max_), area under the plasma concentration-time curve (AUC), half-life (T_1/2_) and mean residence time (MRT).

### 3.8. Acute Toxicity

Male Balb/c mice, aged 6 weeks and weighing 20–22 g, were purchased from the G-Bio (Gyeonggi-do, Korea). The experiments to determine the LD_50_ (lethal dose 50%) of LDH were designed in accordance with the method provided by the OECD (OECD guideline 423). Prior to dosing, food but not water was withheld for 4 h. E-coated CaAl-LDH was homogeneously suspended in 0.9% saline by vortexing and ultrasonicating the suspension for 5 min at 42 kHz (Vibra Cell Sonics & Materials Inc., Newtown, CT, USA). And, four different concentrations of samples (5, 50, 300 and 2000 mg/kg) were administered to each group of three mice via oral gavage. A group of three mice, receiving identical volumes of 0.9% saline, served as a control. Survival rate, body weight change, behaviors and symptoms of mice were carefully recorded daily after treatment.

### 3.9. Statistical Analysis

The data are presented as means ± standard deviations. For statistical analysis, experimental values were compared between experimental groups. One-way analysis of variance (ANOVA) in SAS software (Tukey’s Test, Version 11.0, SAS Institute Inc., Cary, NC, USA) was used to determine the significances of difference between groups. Statistical significance was accepted for *p* values of less than 0.05.

## 4. Conclusions

We demonstrated the potential of polymer-coated CaAl-LDH as a calcium supplement. The pristine CaAl-LDH was well prepared through a simple coprecipitation route, and the coating of CaAl-LDH with enteric polymer Eudragit^®^L 100 (E-coated CaAl-LDH) was carried out in ethanol condition to avoid unintended intercalation of polymer into the LDH interlayer. Physicochemical characterizations revealed that the pristine LDH with hydrocalumite structure was well synthesized and the polymer was effectively coated on the LDH surface with minimal content. Moreover, the dissolution of calcium ions from E-coated CaAl-LDH at pH 1.2 was strongly suppressed compared with the uncoated one. Both pristine CaAl-LDH and E-coated CaAl-LDH did not cause cytotoxic effects on human epithelial colorectal cells, while a remarkably increased calcium level was detected in the nanomaterial-treated cells. Pharmacokinetic analysis clearly demonstrated oral absorption efficiency of E-coated CaAl-LDH, without causing any acute toxicity *in vivo*. It was therefore concluded that CaAl-LDHs with an appropriate coating has great potential as a calcium supplement. 
